# Multiplicity and asymptotic behavior of solutions to a class of Kirchhoff-type equations involving the fractional *p*-Laplacian

**DOI:** 10.1186/s13660-018-1708-9

**Published:** 2018-05-10

**Authors:** Liejun Shen

**Affiliations:** Hubei Key Laboratory of Mathematical Sciences and School of Mathematics and Statistics, Central China Normal University, Wuhan, P.R. China

**Keywords:** 35J47, 35J60, Critical Sobolev exponent, Fractional *p*-Laplacian, Kirchhoff, Multiplicity, Asymptotic behavior

## Abstract

The present study is concerned with the following fractional *p*-Laplacian equation involving a critical Sobolev exponent of Kirchhoff type:

$$\biggl[a+b \biggl( \int_{\mathbb {R}^{2N}}\frac{|u(x)-u(y)|^{p}}{|x-y|^{N+ps}}\,dx\,dy \biggr)^{\theta-1} \biggr](-\Delta)_{p}^{s}u =|u|^{p_{s}^{*}-2}u+\lambda f(x)|u|^{q-2}u \quad\text{in } \mathbb {R}^{N}, $$

where $a,b>0$, $\theta=(N-ps/2)/(N-ps)$ and $q\in(1,p)$ are constants, and $(-\Delta)_{p}^{s}$ is the fractional *p*-Laplacian operator with $0< s<1<p<\infty$ and $ps< N$. For suitable $f(x)$, the above equation possesses at least two nontrivial solutions by variational method for any $a,b>0$. Moreover, we regard $a>0$ and $b>0$ as parameters to obtain convergent properties of solutions for the given problem as $a\searrow0^{+}$ and $b\searrow0^{+}$, respectively.

## Introduction and main results

In this paper, we consider the following fractional *p*-Laplacian equation involving critical Sobolev exponent of Kirchhoff type:

1.1 $$ \biggl[a+b \biggl( \int_{\mathbb {R}^{2N}}\frac { \vert u(x)-u(y) \vert ^{p}}{ \vert x-y \vert ^{N+ps}}\,dx\,dy \biggr)^{\theta-1} \biggr](-\Delta)_{p}^{s}u = \vert u \vert ^{p_{s}^{*}-2}u+\lambda f(x) \vert u \vert ^{q-2}u\quad\text{in } \mathbb {R}^{N}, $$

where $a,b>0$ and $\theta=(N-ps/2)/(N-ps)$ are constants, $p_{s}^{*}=Np/(N-ps)$ is the critical Sobolev exponent, and $(-\Delta)_{p}^{s}$ is the fractional *p*-Laplacian operator with $0< s<1<q<p<\infty$ and $ps< N$ which, up to normalization factors, works on the Riesz potential as

$$(-\Delta)_{p}^{s}\varphi=2\lim_{\sigma\to0^{+}} \int_{B_{\sigma}^{c}(x)} \frac{ \vert \varphi(x)-\varphi(y) \vert ^{p-2} (\varphi(x)-\varphi(y) )}{ \vert x-y \vert ^{N+ps}}\,dy, $$

where $B_{\sigma}^{c}(x)$ is the complement set in $\mathbb {R}^{N}$ of $B_{\sigma}(x):=\{y\in \mathbb {R}^{N}: \vert y-x \vert <\sigma\}$. As for some recent results on the *p*-Laplacian, we refer to [1–6] and the references therein.

We call Eq. (1.1) a Kirchhoff-type *p*-fractional Schrödinger equation because of the appearance of the term $b(\int _{\mathbb {R}^{2N}} \vert u(x)-u(y) \vert ^{p}/ \vert x-y \vert ^{N+ps}\,dx\,dy)^{\theta-1}$. Indeed, if we choose $p=2$, $s=1$, $N=3$ and let $\vert u \vert ^{p_{s}^{*}-2}u+f(x) \vert u \vert ^{q-2}u=k(x,u)-V(x)u$, then (1.1) transforms to the following classical Kirchhoff-type equation:

1.2 $$ - \biggl(a+ b \int_{\mathbb {R}^{3}} \vert \nabla u \vert ^{2}\,dx \biggr) \Delta u+V(x)u=k(x,u), $$

which is degenerate if $b=0$ and non-degenerate otherwise. Equation (1.2) arises in an interesting physical context. In fact, if we set $V(x)=0$ and replace $\mathbb {R}^{3}$ by a bounded domain $\Omega\subset \mathbb {R}^{3}$ in (1.2), then we get the following Kirchhoff Dirichlet problem:

$$- \biggl(a+ b \int_{\Omega} \vert \nabla u \vert ^{2}\,dx \biggr) \Delta u=k(x,u), $$

which is related to the stationary analog of the equation

$$\rho\frac{\partial^{2}u}{\partial t^{2}}- \biggl(\frac{P_{0}}{h}-\frac {E}{2L} \int_{0}^{L} \biggl\vert \frac{\partial u}{\partial x} \biggr\vert ^{2}\,dx \biggr)\frac{\partial^{2}u}{\partial x^{2}}=0, $$

proposed by Kirchhoff in [7] as an extension of the classical D’Alembert wave equation for free vibrations of elastic strings. This model takes the changes in length of the string produced by transverse vibrations into account. After Lions in his pioneering work [8] presented an abstract functional analysis framework to use for (1.2), this problem has been widely studied in extensive literature such as [9–13]. In view of the above facts, it is reasonable to consider the *p*-fractional Kirchhoff equation.

When $a=1$, $b=0$, $p=2$ and let $\vert u \vert ^{p_{s}^{*}-2}u+f(x) \vert u \vert ^{q-2}u=k(x,u)-V(x)u$, then (1.1) can be reduced to the following fractional Schrödinger equation:

1.3 $$ (-\Delta)^{s} u+V(x)u=k(x,u),\quad x\in \mathbb {R}^{N}, $$

which was used to study the standing wave solutions $\psi (x,t)=u(x)e^{-i\omega t}$ for the equation

$$i\hbar\frac{\partial\psi}{\partial t}=\hbar^{2}(-\Delta)^{\alpha }\psi+W(x) \psi-k(x,\psi),\quad x\in \mathbb {R}^{N}, $$

where *ħ* is the Planck constant, $W:\mathbb {R}^{N}\to \mathbb {R}$ is an external potential and *k* is a suitable nonlinearity. Since the fractional Schrödinger equation appears in problems involving nonlinear optics, plasma physics and condensed matter physics, it is one of the main objects of fractional quantum mechanics. To learn more, the reader can refer to [14–23] and the references therein.

Very recently, great attention has been paid to the study of fractional *p*-Laplacian problems. For example, Pucci–Xiang–Zhang [2] were concerned with the nonhomogeneous Schrödinger equations involving the fractional *p*-Laplacian of Kirchhoff type

1.4 $$ M \biggl( \int_{\mathbb {R}^{2N}}\frac{ \vert u(x)-u(y) \vert ^{p}}{ \vert x-y \vert ^{N+ps}}\,dx\,dy \biggr) (- \Delta)_{p}^{s}u+V(x) \vert u \vert ^{p-2}u=k(x,u)+g(x)\quad \text{in } \mathbb {R}^{N}, $$

where *M* is the so-called Kirchhoff function, $k(x,u)$ satisfies the subcritical growth. They employed the mountain-pass theorem and Ekeland’s variational principle to prove that the existence of at least two solutions for (1.4). In [24], Xiang–Zhang–Zhang studied problem (1.1) with $q=1$ and they obtained infinitely many solutions when $\lambda=0$ and for different $a,b,\theta$. They also proved the existence of multiple solutions for suitable $\lambda>0$. Subsequently, if $k(x,u)+g(x)=\xi \vert u \vert ^{p_{s}^{*}-2}u+\tau f(x) \vert u \vert ^{q-2}u$, Wang–Zhang [25] established the existence of infinitely many solutions which tend to zero for suitable positive parameters *ξ* and *τ* by the Kajikiya version of the symmetric mountain-pass theorem. Some other important and meaningful results on the *p*-fractional Schrödinger equation of Kirchhoff type can be found in [26–30] and the references therein.

Before stating our main results, we introduce some useful notations and definitions. Let $D^{s,p}(\mathbb {R}^{N})$ denote the completion of $C^{\infty}_{0}(\mathbb {R}^{N})$ with respect to the norm

$$\Vert u \Vert = \biggl( \int_{\mathbb {R}^{2N}}\frac { \vert u(x)-u(y) \vert ^{p}}{ \vert x-y \vert ^{N+ps}}\,dx\,dy \biggr)^{\frac{1}{p}} $$

and $C_{c}(\mathbb {R}^{N})=\{u\in C(\mathbb {R}^{N}):\operatorname {supp}u \text{ is a compact subset of } \mathbb {R}^{N}\}$. We write $C_{0}(\mathbb {R}^{N})$ for the closure of $C_{c}(\mathbb {R}^{N})$ with respect to the norm $\vert \eta \vert _{\infty}=\sup_{x\in \mathbb {R}^{N}} \vert \eta(x) \vert $. Since a finite measure on $\mathbb {R}^{N}$ is a continuous linear functional on $C_{0}(\mathbb {R}^{N})$, for a measure *μ* we write

$$\Vert \mu \Vert _{0}=\sup_{\eta\in C_{0}(\mathbb {R}^{N}), \vert \eta \vert _{\infty}=1} \bigl\vert ( \mu,\eta) \bigr\vert =\sup_{\eta\in C_{0}(\mathbb {R}^{N}), \vert \eta \vert _{\infty}=1} \biggl\vert \int_{\mathbb {R}^{N}}\eta \,d\mu \biggr\vert . $$

Throughout this paper we shall denote *C* and $C_{i}$ ($i=1, 2,\ldots$) for various positive constants whose exact value may change from line to line but are not essential to the analysis of the problem. $L^{r}(\mathbb {R}^{N})$ $(1\leq r\leq+\infty)$ is the usual Lebesgue space with the standard norm $\vert u \vert _{r}$. We use “→” and “⇀” to denote the strong and weak convergence in the related function spaces, respectively. Let $(X, \Vert \cdot \Vert )$ be a Banach space with its dual space $(X^{*}, \Vert \cdot \Vert _{*})$, and Ψ be its functional on *X*. The Palais–Smale sequence at level $d\in \mathbb {R}$ ($(PS)_{d}$ sequence in short) corresponding to Ψ satisfies $\Psi(x_{n})\to d$ and $\Psi^{\prime}(x_{n})\to0$ as $n\to\infty$, where $\{x_{n}\}\subset X$.

Motivated by all the work mentioned above, we are interested in the multiplicity and asymptotic behavior of solutions for problem (1.1) whose natural variational functional is given by

$$J(u)=\frac{a}{p} \Vert u \Vert ^{p}+\frac{b}{\theta p} \Vert u \Vert ^{\theta p} -\frac{1}{p_{s}^{*}} \int_{\mathbb {R}^{N}} \vert u \vert ^{p_{s}^{*}}\,dx- \frac{\lambda}{q} \int _{\mathbb {R}^{N}}f(x) \vert u \vert ^{q}\,dx. $$

Note that we can employ the idea used in [31] (or [2]) to prove that $J(u)$ is well defined on $D^{s,p}(\mathbb {R}^{N})$ and of class $C^{1}$. Furthermore, any solution of (1.1) is a critical point of $J(u)$. Hence we obtain the solutions of it by finding the critical point of the functional $J(u)$. To this aim, we assume the following condition:

(F) $f\in L^{{p_{s}^{*}}/{(p_{s}^{*}-q)}}(\mathbb {R}^{N})$ with $f(x)\geq0$ and $f(x)\not \equiv0$.

### Definition 1.1

We say that $u\in D^{s,p}(\mathbb {R}^{N})$ is a (weak) solution of (1.1) if

$$\begin{aligned} &\bigl(a+b \Vert u \Vert ^{(\theta-1)p} \bigr) \int_{\mathbb {R}^{2N}}\frac{ \vert u(x)-u(y) \vert ^{p-2} (u(x)-u(y) ) (v(x)-v(y) )}{ \vert x-y \vert ^{N+ps}}\,dx\,dy \\ &\quad= \int_{\mathbb {R}^{N}} \vert u \vert ^{p_{s}^{*}-2}uv \,dx+\lambda \int_{\mathbb {R}^{N}}f(x) \vert u \vert ^{q-2}uv \,dx \end{aligned}$$

for all $v\in D^{s,p}(\mathbb {R}^{N})$.

Our first result is as follows.

### Theorem 1.2

*Assume* (F) *and* $q\in(1,p)$, *then for any* $a,b>0$ *there exists a constant* $\lambda_{*}> 0$ *such that Eq*. (1.1) *has at least two nontrivial solutions*, $u_{1}$ *and* $u_{2}$, *satisfying*

$$J(u_{2})< 0< J(u_{1}),\quad \forall \lambda\in(0,\lambda_{*}). $$

### Remark 1.3

We point out here that if $q=1$ in (1.1), the results in Theorem 1.2 can be seen as a part of [24]. Although the generalization in this sense is trivial, the main interest of this paper is not here, but more attention is paid to the relation between the solutions obtained in Theorem 1.2 and the parameters $a>0$ and $b>0$, and the convergent properties (see Theorems 1.5 and 1.7 below) of the solutions are given. Also, our results extend the results of [32] to fractional Kirchhoff type. Briefly speaking, if $a=s=1$, $b=0$ and $p\geq2$ in (1.1), the results in Theorem 1.2 can be found in [32].

### Remark 1.4

When the nontrivial solutions of (1.1) are obtained, we can prove that the existence of ground state solutions of it. In fact, with Theorem 1.2 in hand, we know that $\mathcal{N}= \{u\in D^{s,p}(\mathbb {R}^{N})\backslash\{0\}:\langle J^{\prime}(u),u\rangle=0 \}\neq\emptyset$ and $m=\inf_{u\in\mathcal{N}}J(u)$ are well defined. Hence any minimizing sequence of *m* is bounded, then by Lemmas 2.6–2.7 below we derive that *m* is attained by some function and it is a ground state solution.

It is worth mentioning that the idea of proving the asymptotic behavior of solutions to (1.1) comes from [12, 33]. Since the solutions $u_{1}$ and $u_{2}$ obtained in Theorem 1.2 depend on the parameter *b*, we next denote $u_{1}$ and $u_{2}$ by $u^{1}_{b}$ and $u_{b}^{2}$ to emphasize this dependence, respectively. By analyzing the convergence property of $u^{1}_{b}$ and $u_{b}^{2}$ as $b\to0^{+}$, we establish one of the following main results in this paper.

### Theorem 1.5

*Assume* (F) *and* $q\in(1,p)$, *let* $\lambda\in(0,\lambda_{*})$ *and* $a>0$ *be fixed constants*, *if* $\{u_{b}^{1}\}$ *and* $\{u_{b}^{2}\}$ *are nontrivial solutions of* (1.1) *obtained in Theorem *1.2, *there exist subsequences still denoted by themselves* $\{ u_{b}^{1}\}$ *and* $\{u_{b}^{2}\}$ *such that* $u_{b}^{i}\to u^{i}$ *in* $D^{s,p}(\mathbb {R}^{N})$ *as* $b\searrow0^{+}$ *for* $i\in\{1,2\}$, *where* $u^{1}$ *and* $u^{2}$ *are two nontrivial solutions of*

1.5 $$ a(-\Delta)_{p}^{s}u= \vert u \vert ^{p_{s}^{*}-2}u+\lambda f(x) \vert u \vert ^{q-2}u \quad\textit{in } \mathbb {R}^{N}. $$

### Remark 1.6

If the whole space $\mathbb {R}^{N}$ is replaced with a bounded domain Ω and assume $b>0$ suffciently small, Lei–Liu–Guo [13] proved that problem (1.1) admits at least two nontrivial solutions when $p=2$, $s=1$, $N=3$ and $f(x)\equiv1$. In a more general case, Theorem 1.5 tells us that the solutions of problem (1.1) are actually the solutions of problem (1.5) if the positive parameter *b* is small enough.

Inspired by Theorem 1.5, the solutions of problem (1.1) also depend on the parameter $a>0$ and then we have the following result.

### Theorem 1.7

*Assume* (F) *and* $q\in(1,p)$, *then there exists* $\lambda _{**}>0$ *such that the problem* (1.1) *admits at least two nontrivial solutions*. *Furthermore if we let* $\lambda\in(0,\lambda _{**})$ *and* $b>0$ *be fixed constants and denote* $\{u_{a}^{11}\}$ *and* $\{ u_{a}^{22}\}$ *are nontrivial solutions of* (1.1) *obtained above*, *then there exist subsequences still denoted by themselves* $\{u_{a}^{11}\}$ *and* $\{u_{a}^{22}\}$ *such that* $u_{a}^{ii}\to u^{ii}$ *in* $D^{s,p}(\mathbb {R}^{N})$ *as* $a\searrow0^{+}$ *for* $i\in\{1,2\}$, *where* $u^{11}$ *and* $u^{22}$ *are two nontrivial solutions of*

1.6 $$ b \biggl( \int_{\mathbb {R}^{2N}}\frac{ \vert u(x)-u(y) \vert ^{p}}{ \vert x-y \vert ^{N+ps}}\,dx\,dy \biggr)^{\theta-1} (- \Delta)_{p}^{s}u = \vert u \vert ^{p_{s}^{*}-2}u+\lambda f(x) \vert u \vert ^{q-2}u \quad\textit{in } \mathbb {R}^{N}. $$

### Remark 1.8

In this paper, we only consider the convergence of the solutions with $a>0$ and $b>0$ as the parameters, respectively. It is natural to raise the following two open problems: (i) Do our results still remain valid when $b<0$ and $b\nearrow0^{-}$*?* (ii) If we take $\lambda>0$ as the parameter and let the positive constants *a* and *b* be fixed, does the convergent property of the solutions still exist when $\lambda\searrow0^{+}$*?* Xiang–Zhang–Zhang [24] studied the existence of solutions for problem (1.1) with $q=1$ and $\lambda=0$, but from our point of view, it seems to be different when it comes to taking $\lambda>0$ as a parameter.

We note that, to the best of our knowledge, there is no result on asymptotic behavior of solutions of critical Kirchhoff-type equations involving the fractional *p*-Laplacian. We now sketch our proofs of Theorems 1.2, 1.5 and 1.7 based on variational method. What makes the proof of Theorem 1.2 more complicated is not only the lack of compactness imbedding of $D^{s,p}(\mathbb {R}^{N})$ into $L^{p_{s}^{*}}(\mathbb {R}^{N})$, but also how to estimate the critical value. To deal with the difficulties mentioned above, some arguments are in order. Using the idea of the well-known Brézis–Nirenberg argument [34], we obtain the threshold value

1.7 $$\begin{aligned} c^{*}={}&a \biggl(\frac{1}{p}- \frac{1}{p_{s}^{*}} \biggr)S^{\frac{N}{ps}} \biggl(\frac{bS^{\frac{2\theta-1}{2}}+\sqrt{b^{2}S^{2\theta -1}+4a}}{2} \biggr)^{\frac{1}{\theta-1}} \\ &{}+b \biggl(\frac{1}{\theta p}-\frac{1}{p_{s}^{*}} \biggr)S^{\frac{N\theta}{ps}} \biggl( \frac{bS^{\frac{2\theta-1}{2}}+\sqrt{b^{2}S^{2\theta -1}+4a}}{2} \biggr)^{\frac{\theta}{\theta-1}} \end{aligned}$$

by solving a quadratic algebra equation with one unknown, where $S>0$ is the best Sobolev constant, that is,

1.8 $$ S=\inf \biggl\{ \int_{\mathbb {R}^{2N}}\frac { \vert u(x)-u(y) \vert ^{p}}{ \vert x-y \vert ^{N+ps}}\,dx\,dy:u\in D^{s,p} \bigl( \mathbb {R}^{N} \bigr) \text{ and } \vert u \vert _{p_{s}^{*}}=1 \biggr\} . $$

After pulling the mountain-pass energy level down below the critical value, we use the celebrated concentration–compactness principle developed by Lions [35] and extended to the fractional Sobolev space $D^{s,p}(\mathbb {R}^{N})$ at some level by Xiang–Zhang–Zhang [24] to show that any (PS) sequence of $J(u)$ contains a strongly convergent subsequence. As to the proof of Theorem 1.5, Although most difficult, the lack of compactness imbedding of $D^{s,p}(\mathbb {R}^{N})$ into $L^{p_{s}^{*}}(\mathbb {R}^{N})$ has been solved, we cannot draw the conclusion that the two sequences of solutions of (1.1) converge to some functions which are nontrivial solutions of (1.5). To overcome it, we have to further estimate the mountain-pass value and local minimum carefully; see (4.3) below for example. Compared with the proof of Theorem 1.5, there are some necessary modifications. For example, Lemma 2.5 below which plays a vital role in the proof Theorem 1.2 can never take positive effect when we take $a\in(0,1]$ as a parameter. Therefore, we can successfully prove Theorems 1.2, 1.5 and 1.7 step by step.

The outline of this paper is as follows. In Sect. 2, we present some preliminary results for Theorem 1.2. In Sect. 3, we obtain the existence of two nontrivial solutions of problem (1.1). In Sects. 4 and 5, we prove the convergent properties on the parameters $b>0$ and $a>0$, respectively.

## Some preliminaries

In this section, we first recall the concentration–compactness principle in the setting of the fractional *p*-Laplacian and then investigate the mountain-pass geometry and the behavior of the $(PS)$ sequence. The following definition can be found in [31].

### Definition 2.1

Let $\mathcal{M}(\mathbb {R}^{N})$ denote the finite nonnegative Borel measure space on $\mathbb {R}^{N}$. For any $\mu\in\mathcal{M}(\mathbb {R}^{N})$, $\mu(\mathbb {R}^{N})= \Vert \mu \Vert _{0}$. We say that $\mu_{n}\rightharpoonup\mu$ weakly ∗ in $\mathcal {M}(\mathbb {R}^{N})$, if $(\mu_{n},\eta)\to(\mu,\eta)$ holds for all $\eta \in C_{0}(\mathbb {R}^{N})$ as $n\to\infty$.

The proofs of the Propositions 2.2–2.4 can be found in [24].

### Proposition 2.2

*Let* $\{u_{n}\}\subset D^{s,p}(\mathbb {R}^{N})$ *with upper bound* $M_{0}> 0$ *for all* $n\geq1$ *and*

$$\begin{aligned} &u_{n}\rightharpoonup u \quad\textit{in } D^{s,p} \bigl( \mathbb {R}^{N} \bigr), \\ &\int_{\mathbb {R}^{N}}\frac{ \vert u_{n}(x)-u_{n}(y) \vert ^{p}}{ \vert x-y \vert ^{N+ps}}\,dy\rightharpoonup \mu\quad \textit{weak } * \textit{ in } \mathcal{M} \bigl(\mathbb {R}^{N} \bigr), \\ &\bigl\vert u_{n}(x) \bigr\vert ^{p_{s}^{*}}\rightharpoonup\nu\quad \textit{weak } * \textit{ in } \mathcal{M} \bigl(\mathbb {R}^{N} \bigr). \end{aligned}$$

*Then*

$$\begin{aligned} &\mu= \int_{\mathbb {R}^{N}}\frac{ \vert u(x)-u(y) \vert ^{p}}{ \vert x-y \vert ^{N+ps}}\,dy+\sum _{j\in {J}}\mu_{j} \delta_{x_{j}}+\overline{ \mu}, \qquad\mu \bigl(\mathbb {R}^{N} \bigr)\leq M_{0}^{p}, \\ &\nu= \vert u \vert ^{p_{s}^{*}}+\sum_{j\in{J}} \nu_{j} \delta_{x_{j}}, \qquad \nu \bigl(\mathbb {R}^{N} \bigr)\leq S^{p_{s}^{*}}M_{0}^{p}, \end{aligned}$$

*where* *J* *is at most countable*, $\{\mu_{j}\},\{\nu_{j}\}$ *are positive constants*, $\{\delta_{x_{j}}\}$ *is the Dirac mass centered at* $x_{j}$, *μ̅* *is a non*-*atomic measure*, $S>0$ *is given by* (1.8) *and*

2.1 $$ \nu \bigl(\mathbb {R}^{N} \bigr)\leq S^{-\frac{p_{s}^{*}}{p}}\mu \bigl( \mathbb {R}^{N} \bigr)^{\frac{p_{s}^{*}}{p}},\qquad \nu_{j}\leq S^{-\frac{p_{s}^{*}}{p}} \mu_{j}^{\frac{p_{s}^{*}}{p}}\quad \textit{for all } j\in J. $$

### Proposition 2.3

*Let* $\{u_{n}\}\subset D^{s,p}(\mathbb {R}^{N})$ *be a bounded sequence such that*

$$\begin{aligned} &\int_{\mathbb {R}^{N}}\frac{ \vert u_{n}(x)-u_{n}(y) \vert ^{p}}{ \vert x-y \vert ^{N+ps}}\,dy\rightharpoonup \mu\quad \textit{weak } * \textit{ in } \mathcal{M} \bigl(\mathbb {R}^{N} \bigr), \\ &\bigl\vert u_{n}(x) \bigr\vert ^{p_{s}^{*}}\rightharpoonup\nu \quad\textit{weak } * \textit{ in } \mathcal{M} \bigl(\mathbb {R}^{N} \bigr), \end{aligned}$$

*and for any* $R>0$ *we define*

$$\begin{aligned} &\mu_{\infty}:=\lim_{R\to\infty}\mathop{\lim \sup}_{n\to\infty } \int_{\{x\in \mathbb {R}^{N}: \vert x \vert >R\}} \int_{\mathbb {R}^{N}}\frac { \vert u_{n}(x)-u_{n}(y) \vert ^{p}}{ \vert x-y \vert ^{N+ps}}\,dy\,dx, \\ &\nu_{\infty}:=\lim_{R\to\infty}\mathop{\lim \sup}_{n\to\infty } \int_{\{x\in \mathbb {R}^{N}: \vert x \vert >R\}} \bigl\vert u_{n}(x) \bigr\vert ^{p_{s}^{*}}\,dx. \end{aligned}$$

*Then the quantities* $\mu_{\infty}$ *and* $\nu_{\infty}$ *are well defined and satisfy*

$$\begin{aligned} &\mathop{\lim\sup}_{n\to\infty} \int_{\mathbb {R}^{2N}}\frac { \vert u_{n}(x)-u_{n}(y) \vert ^{p}}{ \vert x-y \vert ^{N+ps}}\,dy\,dx=\mu \bigl(\mathbb {R}^{N} \bigr)+\mu_{\infty}, \\ &\mathop{\lim\sup}_{n\to\infty} \int_{\mathbb {R}^{N}} \bigl\vert u_{n}(x) \bigr\vert ^{p_{s}^{*}}\,dx=\nu \bigl(\mathbb {R}^{N} \bigr)+\nu_{\infty}. \end{aligned}$$

*Moreover*,

$$\nu_{\infty}\leq S^{-\frac{p}{p_{s}^{*}}}\mu_{\infty}^{\frac{p}{p_{s}^{*}}}. $$

### Proposition 2.4

*Assume that* $\{u_{n}\}\subset D^{s,p}(\mathbb {R}^{N})$ *is the sequence given by Proposition *2.2, *let* $x_{0}\in \mathbb {R}^{N}$ *be fixed and* *ϕ* *be a smooth cut*-*off function such that* $0\leq\phi\leq1$, $\phi \equiv0$ *when* $x\in B_{2}^{c}(0)$, $\phi\equiv1$ *when* $x\in B_{1}(0)$ *and* $\vert \nabla\phi \vert \leq4$. *For any* $\epsilon>0$, *we set* $\phi _{x_{0}}^{\epsilon}(x)=\phi(\frac{x-x_{0}}{\epsilon})$ *for any* $x\in \mathbb {R}^{N}$, *then*

$$\lim_{\epsilon\to0}\mathop{\lim\sup}_{n\to\infty} \int_{\mathbb {R}^{2N}}\frac{ \vert \phi_{x_{0}}^{\epsilon}(x)-\phi_{x_{0}}^{\epsilon }(y) \vert ^{p} \vert u_{n}(x) \vert ^{p}}{ \vert x-y \vert ^{N+ps}}\,dx\,dy=0. $$

Now we will verify that the functional *J* exhibits the mountain-pass geometry.

### Lemma 2.5

*There exists* $\lambda_{0}>0$ *such that the functional* $J(u)$ *satisfies the mountain*-*pass geometry around* $0\in D^{s,p}(\mathbb {R}^{N})$ *for any* $\lambda\in(0, \lambda_{0})$, *that is*,

(i) *there exist* $\alpha,\rho>0$ *such that* $J(u)\geq\alpha>0$ *when* $\Vert u \Vert =\rho$ *and* $\lambda\in(0, \lambda_{0})$;
(ii) *there exists* $e\in D^{s,p}(\mathbb {R}^{N})$ *with* $\Vert e \Vert >\rho$ *such that* $J(e)<0$.

### Proof

(i) It follows from (1.8) and Hölder’s inequality that

$$\begin{aligned} J(u)&\geq \frac{a}{p} \Vert u \Vert ^{p}- \frac{1}{p_{s}^{*}}S^{-\frac{p_{s}^{*}}{p}} \Vert u \Vert ^{p_{s}^{*}} - \frac{\lambda}{q} \vert f \vert _{\frac{p_{s}^{*}}{p_{s}^{*}-q}}S^{-\frac {q}{p}} \Vert u \Vert ^{q} \\ &= \Vert u \Vert ^{q} \biggl(\frac{a}{p} \Vert u \Vert ^{p-q}-\frac{1}{p_{s}^{*}}S^{-\frac {p_{s}^{*}}{p}} \Vert u \Vert ^{p_{s}^{*}-q} -\frac{\lambda}{q} \vert f \vert _{\frac {p_{s}^{*}}{p_{s}^{*}-q}}S^{-\frac{q}{p}} \biggr) \\ &\geq \biggl[\frac{ap_{s}^{*}S^{\frac{p_{s}^{*}}{p}}(p-q)}{p(p_{s}^{*}-q)} \biggr]^{\frac{q}{p_{s}^{*}-p}} \biggl\{ \frac{a(p_{s}^{*}-p)}{p(p_{s}^{*}-q)} \biggl[\frac{ap_{s}^{*}S^{\frac{p_{s}^{*}}{p}}(p-q)}{p(p_{s}^{*}-q)} \biggr]^{\frac {p-q}{p_{s}^{*}-p}} - \frac{\lambda}{q} \vert f \vert _{\frac{p_{s}^{*}}{p_{s}^{*}-q}}S^{-\frac{q}{p}} \biggr\} . \end{aligned}$$

Therefore if we set

$$\rho= \biggl[\frac{ap_{s}^{*}S^{\frac{p_{s}^{*}}{p}}(p-q)}{p(p_{s}^{*}-q)} \biggr]^{\frac{1}{p_{s}^{*}-p}}>0 \quad\text{and}\quad \lambda_{0}=\frac{aqS^{\frac{q}{p}}(p_{s}^{*}-p)}{p \vert f \vert _{\frac {p_{s}^{*}}{p_{s}^{*}-q}}(p_{s}^{*}-q)} \biggl[\frac{ap_{s}^{*}S^{\frac {p_{s}^{*}}{p}}(p-q)}{p(p_{s}^{*}-q)} \biggr]^{\frac{p-q}{p_{s}^{*}-p}} >0, $$

then there exists $\alpha>0$ such that $J(u)\geq\alpha>0$ when $\Vert u \Vert =\rho>0$ for any $\lambda\in(0,\lambda_{0})$.

(ii) Choosing $u_{0}\in D^{s,p}(\mathbb {R}^{N})\backslash\{0\}$, then since $\theta p< p_{s}^{*}$ and $f(x)$ is nonnegative one has

$$J(tu_{0})\leq\frac{a}{p}t^{p} \Vert u_{0} \Vert ^{p}+\frac{b}{\theta p}t^{\theta p} \Vert u_{0} \Vert ^{\theta p}-\frac{t^{p_{s}^{*}}}{p_{s}^{*}} \int_{\mathbb {R}^{N}} \vert u_{0} \vert ^{p_{s}^{*}}\,dx \to-\infty \quad\text{as } t\to+\infty. $$

Hence letting $e=t_{0}u_{0}\in D^{s,p}(\mathbb {R}^{N})\backslash\{0\}$ with $t_{0}$ sufficiently large, we have $\Vert e \Vert >\rho$ and $J(e)<0$. The proof is complete. □

By Lemma 2.5, and the mountain-pass theorem in [31], a $(PS)$ sequence of the functional $J(u)$ at the level

2.2 $$ c:=\inf_{\gamma\in\Gamma}\max_{t\in[0,1]}J \bigl(\gamma(t) \bigr)\geq\alpha>0 $$

can be constructed, where the set of paths is defined as

$$\Gamma:= \bigl\{ \gamma\in C \bigl([0,1],D^{s,p} \bigl(\mathbb {R}^{N} \bigr) \bigr):\gamma(0)=0, J \bigl(\gamma(1) \bigr)< 0 \bigr\} . $$

In other words, there exists a sequence $\{u_{n}\}\subset D^{s,p}(\mathbb {R}^{N})$ such that

2.3 $$ J(u_{n})\to c,\qquad J^{\prime}(u_{n})\to0\quad \text{as } n\to\infty. $$

As the existence of the critical Sobolev exponent in (1.1), we have to estimate the mountain-pass value given by (2.2) carefully. Thanks to the results in [36], there exists a positive function $U(x)$ satisfying

$$(-\Delta)_{p}^{s}u=u^{p_{s}^{*}-1} \quad\text{in } \mathbb {R}^{N} $$

and $\Vert U \Vert ^{p}= \vert U \vert _{p_{s}^{*}}^{p_{s}^{*}}=S^{N/ps}$.

### Lemma 2.6

*There exists* $\lambda_{*}>0$ *such that the mountain*-*pass value satisfies*

$$c< c^{*}-C_{0}\lambda ^{\frac{p}{p-q}}, \quad\textit{where } C_{0}= \frac{a(\theta-1)(p-q)}{q\theta p} \biggl[\frac{(\theta p-q) \vert f \vert _{{p_{s}^{*}}/{(p_{s}^{*}-q)}}}{a(\theta-1)pS^{\frac{q}{p}}} \biggr]^{\frac{p}{p-q}} >0 $$

*for any* $\lambda\in(0,\lambda_{*})$, $c^{*}$ *and* *S* *are given by* (1.7) *and* (1.8), *respectively*.

### Proof

It is obvious that there exists $\lambda_{1}>0$ independent of *b* such that

$$c^{*}-C_{0}\lambda ^{\frac{p}{p-q}}>0 \quad\text{for any } \lambda\in(0, \lambda_{1}). $$

We then claim that

2.4 $$ J(tU)\leq c^{*} \quad\text{for any } t\geq0. $$

Indeed, let us define

$$g(t)=\frac{a}{p}t^{p} \Vert U \Vert ^{p}+ \frac{b}{\theta p}t^{\theta p} \Vert U \Vert ^{\theta p}- \frac{t^{p_{s}^{*}}}{p_{s}^{*}} \int_{\mathbb {R}^{N}} \vert U \vert ^{p_{s}^{*}}\,dx:= C_{1}t^{p}+C_{2}t^{\theta p}-C_{3}t^{p_{s}^{*}},\quad t\geq0, $$

where

$$\begin{aligned} &C_{1}=\frac{a}{p} \Vert U \Vert ^{p}= \frac{a}{p}S^{\frac{N}{ps}},\qquad C_{2}=\frac {b}{\theta p} \Vert U \Vert ^{\theta p}=\frac{b}{\theta p}S^{\frac{N\theta }{ps}}, \\ & C_{3}= \frac{1}{p_{s}^{*}} \int_{\mathbb {R}^{N}} \vert U \vert ^{p_{s}^{*}}\,dx= \frac {1}{p_{s}^{*}}S^{\frac{N}{ps}}. \end{aligned}$$

By some elementary calculations, we have

$$g^{\prime}(t)=C_{1}p t^{p-1}+C_{2}\theta pt^{\theta p-1}-C_{3}p_{s}^{*}t^{p_{s}^{*}-1}=0,\quad t\geq0, $$

which is equivalent to

$$C_{1}p+C_{2}\theta pt^{\theta p-p}-C_{3}p_{s}^{*}t^{p_{s}^{*}-p}=0,\quad t\geq0. $$

Since $p_{s}^{*}-p=2(\theta p-p)$, we know that $g^{\prime}(t)=0$ has a unique root, that is,

$$t_{0}= \biggl(\frac{C_{2}\theta p+\sqrt{C_{2}^{2}\theta^{2} p^{2}+4C_{1}C_{3}p_{s}^{*}p}}{2C_{3}p_{s}^{*}} \biggr)^{\frac{1}{\theta p-p}} = \biggl( \frac{bS^{\frac{2\theta-1}{2}}+\sqrt{b^{2}S^{2\theta -1}+4a}}{2} \biggr)^{\frac{1}{\theta p-p}}>0, $$

where we use the fact that $\theta=(N-ps/2)/(N-ps)$. Therefore we can conclude that

2.5 $$\begin{aligned} \max_{t\geq0}g(t) &=g(t_{0})=C_{1}t_{0}^{p}+C_{2}t_{0}^{\theta p}- \frac {C_{1}p t_{0}^{p}+C_{2}\theta p t_{0}^{\theta p}}{p_{s}^{*}} \\ & = a \biggl(\frac{1}{p}-\frac{1}{p_{s}^{*}} \biggr)S^{\frac{N}{ps}}t_{0}^{p} +b \biggl(\frac{1}{\theta p}-\frac{1}{p_{s}^{*}} \biggr)S^{\frac{N\theta }{ps}}t_{0}^{\theta p}=c^{*}, \end{aligned}$$

which together with the fact $f(x)$ is nonnegative gives (2.4).

Since $J(0)=0$, there exists $t_{1}\in(0,1)$ such that

$$\max_{0\leq t\leq t_{1}}J(tU)< c^{*}-C_{0}\lambda ^{\frac{p}{p-q}} \quad\text{for any } \lambda\in(0,\lambda_{1}). $$

On the other hand, the facts

$$J(tU)=g(t)-\frac{t^{q}}{q}\lambda \int_{\mathbb {R}^{N}}f(x) \vert U \vert ^{q}\,dx \leq\max _{t\geq0}g(t)-\frac{t^{q}}{q}\lambda \int_{\mathbb {R}^{N}}f(x) \vert U \vert ^{q}\,dx $$

and (2.5) show that

$$\max_{t\geq t_{1}}J(tU)\leq c^{*}-\frac{t_{1}^{q}}{q}\lambda \int_{\mathbb {R}^{3}}f(x) \vert U \vert ^{q}\,dx. $$

Taking

$$\lambda_{2}= \biggl(\frac{t_{1}^{q}\int_{\mathbb {R}^{N}}f(x) \vert U \vert ^{q}\,dx}{C_{0}q} \biggr)^{\frac{p-q}{q}}>0, $$

then we have

$$\max_{t\geq t_{1}}J(tU)< c^{*}-C_{0}\lambda^{\frac{p}{p-q}} \quad\text{for any } 0< \lambda< \lambda_{2}. $$

Finally, choosing $\lambda_{*}=\min\{\lambda_{0},\lambda_{1},\lambda_{2}\} >0$ we can deduce that

$$\max_{t\geq0}J(tU)< c^{*}-C_{0}\lambda^{\frac{p}{p-q}} \quad\text{for any } 0< \lambda< \lambda_{*}, $$

which yields the proof of this lemma. □

The following lemma provides the interval where the $(PS)$ condition holds for $J(u)$.

### Lemma 2.7

*If* $\lambda\in(0,\lambda_{*})$, *any sequence satisfying* (2.2) *contains a strongly convergent subsequence whenever* $c< c^{*}-C_{0}\lambda^{\frac{p}{p-q}}$, *where* $c^{*}$ *is given by* (1.7).

### Proof

Let $\{u_{n}\}\subset D^{s,p}(\mathbb {R}^{N})$ be a sequence verifying (2.3) and we conclude that $\{u_{n}\}$ is bounded in $D^{s,p}(\mathbb {R}^{N})$. Recalling that $\theta=(N-ps/2)/(N-ps)>1$, then we have

$$\begin{aligned} c+1+o(1) \Vert u_{n} \Vert &\geq J(u_{n})- \frac{1}{\theta p} \bigl\langle J^{\prime }(u_{n}),u_{n} \bigr\rangle \\ &\geq a \biggl(\frac{1}{p}-\frac{1}{\theta p} \biggr) \Vert u_{n} \Vert ^{p}- \biggl(\frac{1}{q}- \frac{1}{\theta p} \biggr)\lambda \vert f \vert _{\frac {p_{s}^{*}}{p_{s}^{*}-q}}S^{-\frac{q}{p}} \Vert u_{n} \Vert ^{q}, \end{aligned}$$

which shows that $\{u_{n}\}$ is bounded in $D^{s,p}(\mathbb {R}^{N})$ since $p>q>1$. Up to a subsequence if necessary, there exists $u\in D^{s,p}(\mathbb {R}^{N})$ such that $u_{n}\rightharpoonup u$ in $D^{s,p}(\mathbb {R}^{N})$, $u_{n}\to u$ in $L^{r}_{\text{loc}}(\mathbb {R}^{N})$ for $r\in[1,p_{s}^{*})$ and $u_{n}\to u$ a.e. in $\mathbb {R}^{N}$. Obviously, the conclusions in Proposition 2.2 are true in the sense of a subsequence. Now we prove that $u_{n}\to u$ in $D^{s,p}(\mathbb {R}^{N})$.

To do it, we first claim that the set *J* given by Proposition 2.2 is an empty set. Arguing it by contradiction, for some $j\in J$ and for any $\epsilon >0$ choosing $\phi_{j}^{\epsilon}$ to be a smooth cut-off function such that $0\leq\phi_{j}^{\epsilon}\leq1$, $\phi_{j}^{\epsilon}\equiv0$ when $x\in B_{\epsilon}^{c}(x_{j})$, $\phi_{j}^{\epsilon}\equiv1$ when $x\in B_{{\epsilon}/{2}}(x_{j})$ and $\vert \nabla\phi_{j}^{\epsilon} \vert \leq {4}/{\epsilon}$. It follows from Proposition 2.2 that

$$\begin{aligned} & \lim_{\epsilon\to0}\lim _{n\to\infty} \bigl(a+b \Vert u_{n} \Vert ^{(\theta -1)p} \bigr) \int_{\mathbb {R}^{2N}} \frac{ \vert u_{n}(x)-u_{n}(y) \vert ^{p}\phi_{j}^{\epsilon}(y)}{ \vert x-y \vert ^{N+ps}}\,dx\,dy \\ &\quad\geq\lim_{\epsilon\to0}\lim_{n\to\infty} \biggl[a \int_{\mathbb {R}^{2N}} \frac{ \vert u_{n}(x)-u_{n}(y) \vert ^{p}\phi_{j}^{\epsilon}(y)}{ \vert x-y \vert ^{N+ps}}\,dx\,dy \\ &\qquad{}+b \biggl( \int_{\mathbb {R}^{2N}} \frac{ \vert u_{n}(x)-u_{n}(y) \vert ^{p}\phi_{j}^{\epsilon}(y)}{ \vert x-y \vert ^{N+ps}}\,dx\,dy \biggr)^{\theta} \biggr] \\ &\quad=a\mu_{j}+b\mu_{j}^{\theta} \end{aligned}$$

and

$$\begin{aligned} &\biggl\vert \bigl(a+b \Vert u_{n} \Vert ^{(\theta-1)p} \bigr) \int_{\mathbb {R}^{2N}} \frac{ \vert u_{n}(x)-u_{n}(y) \vert ^{p-2} (u_{n}(x)-u_{n}(y) ) (\phi_{j}^{\epsilon}(x)-\phi_{j}^{\epsilon}(y) )u_{n}(x)}{ \vert x-y \vert ^{N+ps}}\,dx\,dy \biggr\vert \\ &\quad\leq C \biggl( \int_{\mathbb {R}^{2N}} \frac { \vert u_{n}(x)-u_{n}(y) \vert ^{p}}{ \vert x-y \vert ^{N+ps}}\,dx\,dy \biggr)^{\frac{p-1}{p}} \biggl( \int_{\mathbb {R}^{2N}} \frac{ \vert \phi_{j}^{\epsilon}(x)-\phi_{j}^{\epsilon}(y) \vert ^{p} \vert u_{n}(x) \vert ^{p}}{ \vert x-y \vert ^{N+ps}}\,dx\,dy \biggr)^{\frac{1}{p}} \\ &\quad\leq C \biggl( \int_{\mathbb {R}^{2N}} \frac{ \vert \phi_{j}^{\epsilon}(x)-\phi _{j}^{\epsilon}(y) \vert ^{p} \vert u_{n}(x) \vert ^{p}}{ \vert x-y \vert ^{N+ps}}\,dx\,dy \biggr)^{\frac {1}{p}} \to0\quad \text{as } \epsilon\to0 \text{ and } n\to\infty, \end{aligned}$$

where we have used Proposition 2.4. We also know that

$$\lim_{\epsilon\to0}\lim_{n\to\infty} \int_{\mathbb {R}^{N}} \vert u_{n} \vert ^{p_{s}^{*}} \phi_{j}^{\epsilon}\,dx=\lim_{\epsilon\to0}\lim _{n\to \infty} \int_{B_{\epsilon}(x_{j})} \vert u_{n} \vert ^{p_{s}^{*}} \phi_{j}^{\epsilon}\,dx+\nu _{j}=\nu_{j} $$

and

$$\lim_{\epsilon\to0}\lim_{n\to\infty} \int_{\mathbb {R}^{N}}f(x) \vert u_{n} \vert ^{q} \phi _{j}^{\epsilon}\,dx=\lim_{\epsilon\to0}\lim _{n\to\infty} \int _{B_{\epsilon}(x_{j})}f(x) \vert u_{n} \vert ^{q} \phi_{j}^{\epsilon}\,dx=0. $$

Since $u_{n}\phi_{j}^{\epsilon}\in D^{s,p}(\mathbb {R}^{N})$ is bounded, we have $\langle J^{\prime}(u_{n}),u_{n}\phi_{j}^{\epsilon}\rangle=o(1)$, that is,

$$\begin{aligned} &\bigl(a+b \Vert u_{n} \Vert ^{(\theta-1)p} \bigr) \int_{\mathbb {R}^{2N}}\frac{ \vert u_{n}(x)-u_{n}(y) \vert ^{p-2} (u_{n}(x)-u_{n}(y) ) (u_{n}(x)\phi_{j}^{\epsilon}(x)-u_{n}(y)\phi_{j}^{\epsilon}(y) )}{ \vert x-y \vert ^{N+ps}}\,dx\,dy \\ &\quad= \int_{\mathbb {R}^{N}} \vert u_{n} \vert ^{p_{s}^{*}} \phi_{j}^{\epsilon}\,dx+ \int_{\mathbb {R}^{N}}f(x) \vert u_{n} \vert ^{q} \phi_{j}^{\epsilon}\,dx+o(1). \end{aligned}$$

It is easy to see that

$$\begin{aligned} & \bigl\vert u_{n}(x)-u_{n}(y) \bigr\vert ^{p-2} \bigl(u_{n}(x)-u_{n}(y) \bigr) \bigl(u_{n}(x)\phi _{j}^{\epsilon}(x)-u_{n}(y) \phi_{j}^{\epsilon}(y) \bigr) \\ &\quad= \bigl\vert u_{n}(x)-u_{n}(y) \bigr\vert ^{p}\phi_{j}^{\epsilon}(y) \\ &\qquad{}+ \bigl\vert u_{n}(x)-u_{n}(y) \bigr\vert ^{p-2} \bigl(u_{n}(x)-u_{n}(y) \bigr) \bigl( \phi_{j}^{\epsilon}(x)-\phi_{j}^{\epsilon}(y) \bigr)u_{n}(x). \end{aligned}$$

Coming the above six formulas, we have $\nu_{j}\geq a\mu_{j}+b\mu _{j}^{\theta}$. In view of (2.1) and $p_{s}^{*}/p=2\theta-1$, we obtain

$$S^{-(2\theta-1)}\mu_{j}^{2(\theta-1)}-b\mu_{j}^{\theta-1}-a \geq0, $$

which gives that

$$\mu_{j}\geq \biggl(\frac{b+\sqrt{b^{2}+4aS^{-(2\theta -1)}}}{2S^{-(2\theta-1)}} \biggr)^{\frac{1}{\theta-1}} =S^{\frac {N}{ps}} \biggl(\frac{bS^{\frac{2\theta-1}{2}}+\sqrt{b^{2}S^{2\theta -1}+4a}}{2} \biggr)^{\frac{1}{\theta-1}}. $$

Using Proposition 2.2 and (2.3) again, we derive

2.6 $$\begin{aligned} c+o(1)={}&J(u_{n})-\frac{1}{\theta p} \bigl\langle J^{\prime }(u_{n}),u_{n} \bigr\rangle \\ ={}&a \biggl(\frac{1}{p}-\frac{1}{\theta p} \biggr) \Vert u_{n} \Vert ^{p}+ \biggl(\frac{1}{\theta p}- \frac{1}{ p_{s}^{*}} \biggr) \int_{\mathbb {R}^{N}} \vert u_{n} \vert ^{p_{s}^{*}} \,dx \\ &{}- \biggl(\frac{1}{q}-\frac{1}{\theta p} \biggr)\lambda \int_{\mathbb {R}^{N}}f(x) \vert u_{n} \vert ^{q} \,dx \\ \geq{}& a \biggl(\frac{1}{p}-\frac{1}{\theta p} \biggr) \bigl(\mu _{j}+ \Vert u \Vert ^{p} \bigr)+ \biggl( \frac{1}{\theta p}-\frac{1}{ p_{s}^{*}} \biggr)\nu _{j}- \biggl( \frac{1}{q}-\frac{1}{\theta p} \biggr)\lambda \vert f \vert _{\frac {p_{s}^{*}}{p_{s}^{*}-q}}S^{-\frac{q}{p}} \Vert u \Vert ^{q} \\ \geq{}& a \biggl(\frac{1}{p}-\frac{1}{\theta p} \biggr) \mu _{j} + \biggl(\frac{1}{\theta p}-\frac{1}{ p_{s}^{*}} \biggr) \bigl(a \mu_{j}+b\mu _{j}^{\theta} \bigr)-C_{0} \lambda^{\frac{p}{p-q}} \\ ={}&a \biggl(\frac{1}{p}-\frac{1}{p_{s}^{*}} \biggr) \mu_{j}+b \biggl(\frac {1}{\theta p}-\frac{1}{ p_{s}^{*}} \biggr)\mu_{j}^{\theta}-C_{0} \lambda ^{\frac{p}{p-q}} \geq c^{*}-C_{0}\lambda^{\frac{p}{p-q}}, \end{aligned}$$

a contradiction. Hence we have $J=\emptyset$.

We then claim that the quantities $\mu_{\infty}$ and $\nu_{\infty}$ given by Proposition 2.3 satisfy $\mu_{\infty}=\nu _{\infty}\equiv0$. For any $R>0$, let $\varphi_{R}(x)$ to be a smooth function such that $0\leq\varphi_{R}\leq1$, $\varphi_{R}\equiv1$ when $x\in B_{R}^{c}(0)$, $\varphi_{R}\equiv0$ when $x\in B_{R/{2}}(0)$ and $\vert \nabla\varphi_{R} \vert \leq{4}/R$. Now repeating the same process of proving the above claim, we can obtain $\mu_{\infty}=\nu_{\infty}\equiv0$.

Finally, based on the above two claims and [24, Lemma 4.5], we have $u_{n}\to u$ in $D^{s,p}(\mathbb {R}^{N})$. Therefore $J^{\prime}(u)=0$ and $J(u)=c$. The proof is complete. □

## The proof of Theorem 1.2

In this section, we will prove Theorem 1.2 in detail.

### Existence of a first solution for (1.1)

#### Proof

Let $\lambda _{*}>0$ be given as in Lemma 2.6, then for any $\lambda\in(0,\lambda_{*})$ there exists a sequence $\{u_{n}\}\subset D^{s,p}(\mathbb {R}^{N})$ verifying (2.3) by Lemma 2.5. In view of the proof of Lemma 2.7, we know that there exists a critical point $u_{1}\in D^{s,p}(\mathbb {R}^{N})$ of *J* such that $J(u_{1})=c>0$. Hence $u_{1}$ is a nontrivial solution of (1.1). □

### Existence of a second solution for (1.1)

Before we obtain the second solution, we introduce the following proposition.

#### Proposition 3.1

(Ekeland’s variational principle [37], Theorem 1.1)

*Let* *V* *be a complete metric space and* $F:V\to \mathbb {R}\cup\{+\infty\}$ *be lower semicontinuous*, *bounded from below*. *Then*, *for any* $\epsilon>0$, *there exists some point* $v\in V$ *with*

$$F(v)\leq\inf_{V}F+\epsilon,\qquad F(w)\geq F(v)-\epsilon d(v,w) \quad\textit {for all } w\in V. $$

We are in a position to show the existence of a second positive solution for (1.1).

#### Proof

For $\rho>0$ given by Lemma 2.5(i), we define

$$\overline{B}_{\rho}= \bigl\{ u\in D^{s,p} \bigl( \mathbb {R}^{N} \bigr), \Vert u \Vert \leq\rho \bigr\} ,\qquad \partial B_{\rho}= \bigl\{ u\in D^{s,p} \bigl(\mathbb {R}^{N} \bigr), \Vert u \Vert = \rho \bigr\} , $$

and clearly $\overline{B}_{\rho}$ is a complete metric space with the distance $d(u,v)= \Vert u-v \Vert $. It is obvious that the functional *J* is lower semicontinuous and bounded from below on $\overline{B}_{\rho}$ (see [31]).

We claim first that

3.1 $$ \widetilde{c} :=\inf \bigl\{ J(u):u\in\overline{B}_{\rho}\bigr\} < 0. $$

Indeed, choosing a nonnegative function $\psi\in C_{0}^{\infty}(\mathbb {R}^{N})$ and then we have

$$\lim_{t\to0}\frac{J(t\psi)}{t^{q}}=-\frac{\lambda}{q} \int_{\mathbb {R}^{N}}f(x) \vert \psi \vert ^{q}\,dx< 0. $$

Therefore there exists a sufficiently small $t_{0}>0$ such that $\Vert t_{0}\psi \Vert \leq\rho$ and $J(t_{0}\psi)<0$, which imply that (3.1) holds. By Proposition 3.1, for any $n\in N$ there exists $\widetilde{u}_{n}$ such that

3.2 $$ \widetilde{ c}\leq J(\widetilde{u}_{n})\leq \widetilde{c} +\frac{1}{n} \quad\text{and}\quad J(v)\geq J(\widetilde{u}_{n})- \frac{1}{n} \Vert \widetilde{u}_{n}-v \Vert , \quad\forall v\in \overline{B}_{\rho}. $$

Then we claim that $\Vert \widetilde{u}_{n} \Vert <\rho$ for $n\in N$ sufficiently large. In fact, we will argue it by contradiction and just suppose that $\Vert \widetilde{u}_{n} \Vert =\rho$ for infinitely many *n*, without loss of generality, we may assume that $\Vert \widetilde{u}_{n} \Vert =\rho$ for any $n\in N$. It follows from Lemma 2.5 that $J(\widetilde{u}_{n})\geq\alpha>0$ and by (3.2) we have $\widetilde{c}\geq\alpha>0$ which is a contradiction to (3.1). Next, we will show that $J^{\prime}(\widetilde{u}_{n})\to0$ in $(D^{s,p}(\mathbb {R}^{N}))^{*}$. Indeed, set

$$v_{n}=\widetilde{u}_{n}+tu,\quad \forall u\in B_{1}= \bigl\{ u\in D^{s,p} \bigl(\mathbb {R}^{N} \bigr), \Vert u \Vert =1 \bigr\} , $$

where $t>0$ small enough such that $0< t\leq\rho- \Vert \widetilde{u}_{n} \Vert $ for fixed *n* large, then

$$\Vert v_{n} \Vert = \Vert \widetilde{u}_{n}+tu \Vert \leq \Vert \widetilde{u}_{n} \Vert +t\leq\rho, $$

which imply that $v_{n}\in\overline{B}_{\rho}$. So it follows from (3.2) that

$$J(v_{n})\geq J(\widetilde{u}_{n})-\frac{t}{n} \Vert \widetilde{u}_{n}-v_{n} \Vert , $$

that is,

$$\frac{J(\widetilde{u}_{n}+tu)-J(\widetilde{u}_{n})}{t}\geq-\frac{1}{n}. $$

Letting $t\to0$, then we have $\langle J^{\prime}(\widetilde{u}_{n}), u\rangle\geq-\frac{1}{n}$ for any fixed *n* large. Similarly, choosing $t<0$ and $\vert t \vert $ small enough, and repeating the process above we have $\langle J^{\prime}(\widetilde{u}_{n}), u\rangle\leq\frac {1}{n}$ for any fixed *n* large. Therefore the conclusion $\langle J^{\prime}(\widetilde{u}_{n}), u\rangle\to0$ as $n\to\infty $ for any $u\in B_{1}$ implies that $J^{\prime}(\widetilde{u}_{n})\to0$ in $(D^{s,p}(\mathbb {R}^{N}))^{*}$.

Hence, we know that $\{\widetilde{u}_{n}\}$ is a $(PS)_{\widetilde{c}}$ sequence for the functional $J(u)$ with $\widetilde{c}<0$. Therefore, Lemma 2.7 implies that there exists a function $u_{2}\in \overline{B}_{\rho}$ such that $J^{\prime}(u_{2})=0$ and $J(u_{2})=\widetilde{c}<0$. Hence $u_{2}$ is a nontrivial solution of (1.1). □

## Asymptotic behavior as $b\searrow0^{+}$

In this section, we prove Theorem 1.5. In the following, we regard $b>0$ as a parameter in problem (1.1) and analyze the convergence property of $u_{b}^{i}$ as $b\searrow0$ for $i\in\{1,2\}$. The variational functional corresponding to (1.5) is given by

$$J_{0}(u)=\frac{a}{p} \Vert u \Vert ^{p}- \frac{1}{p_{s}^{*}} \int_{\mathbb {R}^{N}} \vert u \vert ^{p_{s}^{*}}\,dx- \frac{\lambda}{q} \int_{\mathbb {R}^{N}}f(x) \vert u \vert ^{q}\,dx, $$

which is of class of $C^{1}$ due to [31] (or [2]). For any $b\in(0,1]$, we have

$$\begin{aligned} c^{*}\leq{}& a \biggl(\frac{1}{p}- \frac{1}{p_{s}^{*}} \biggr)S^{\frac{N}{ps}} \biggl(\frac{S^{\frac{2\theta-1}{2}}+\sqrt{S^{2\theta -1}+4a}}{2} \biggr)^{\frac{1}{\theta-1}} \\ &{}+ \biggl(\frac{1}{\theta p}-\frac{1}{p_{s}^{*}} \biggr)S^{\frac{N\theta}{ps}} \biggl( \frac{S^{\frac{2\theta-1}{2}}+\sqrt{S^{2\theta -1}+4a}}{2} \biggr)^{\frac{\theta}{\theta-1}} :=M< +\infty, \end{aligned}$$

where *M* is independent of *b*. Let $\{u_{b}^{i}\}$ ($i\in\{1,2\}$) be the solutions of (1.1) obtained in Theorem 1.2, that is,

4.1 $$ J^{\prime}_{b} \bigl(u_{b}^{1} \bigr)=0,\qquad J_{b} \bigl(u_{b}^{1} \bigr)=c_{b} $$

and

4.2 $$ J^{\prime}_{b} \bigl(u_{b}^{2} \bigr)=0, \qquad J_{b} \bigl(u_{b}^{2} \bigr)= \widetilde{c_{b}}, $$

where

$$J_{b}(u)=\frac{a}{p} \Vert u \Vert ^{p}+ \frac{b}{\theta p} \Vert u \Vert ^{\theta p} -\frac{1}{p_{s}^{*}} \int_{\mathbb {R}^{N}} \vert u \vert ^{p_{s}^{*}}\,dx- \frac{\lambda}{q} \int _{\mathbb {R}^{N}}f(x) \vert u \vert ^{q}\,dx. $$

### Proof of Theorem 1.5

To present the proof clearly, we will split it into several steps:

*Step* 1: there exist four constants independent of $b\in (0,1]$ such that

4.3 $$ 0< \alpha\leq c_{b}< M-C_{0} \lambda^{\frac{p}{p-q}} \quad\text{and}\quad {-}C_{0}\lambda^{\frac{p}{p-q}}\leq \widetilde{c_{b}}\leq-\frac{\lambda }{2q} \int_{\mathbb {R}^{N}}f(x) \vert \psi_{0} \vert ^{q}\,dx< 0. $$

In fact, the constant $\alpha>0$ given by Lemma 2.5 is independent of any $b>0$, then by (2.2) we have $J_{b}(u_{b}^{1})\geq\alpha$. On the other hand, using (4.2) we have

$$\begin{aligned} J_{b} \bigl(u_{b}^{2} \bigr)&=J_{b} \bigl(u_{b}^{2} \bigr)-\frac{1}{\theta p} \bigl\langle J^{\prime }_{b} \bigl(u_{b}^{2} \bigr),u_{b}^{2} \bigr\rangle \\ &\geq a \biggl(\frac{1}{p}-\frac{1}{\theta p} \biggr) \bigl\Vert u_{b}^{2} \bigr\Vert ^{p}- \biggl( \frac{1}{q}-\frac{1}{\theta p} \biggr)\lambda \vert f \vert _{\frac {p_{s}^{*}}{p_{s}^{*}-q}}S^{-\frac{q}{p}} \bigl\Vert u_{b}^{2} \bigr\Vert ^{q}\geq-C_{0}\lambda^{\frac {p}{p-q}}, \end{aligned}$$

where $C_{0}>0$ is given by Lemma 2.6. We choose a nonnegative function $\psi_{0}\in C_{0}^{\infty}(\mathbb {R}^{N})$ to satisfy $\Vert \psi_{0} \Vert \leq(2qC_{0}/ \vert f \vert _{{p_{s}^{*}}/{(p_{s}^{*}-q)}})^{\frac{1}{q}}\lambda ^{\frac{1}{p-q}}S^{\frac{1}{p}}$. Since

$$\lim_{t\to0}\frac{J_{b}(t\psi_{0})}{t^{q}}=-\frac{\lambda}{q} \int _{\mathbb {R}^{N}}f(x) \vert \psi_{0} \vert ^{q}\,dx< 0, $$

we can let $t_{0}>0$ such that $\Vert t_{0}\psi \Vert \leq\rho$, where $\rho>0$ is given by Lemma 2.5(ii). Therefore we can obtain

$$\widetilde{c_{b}}=\inf \bigl\{ J_{b}(u):u\in \overline{B}_{\rho}\bigr\} \leq-\frac{\lambda}{2q} \int_{\mathbb {R}^{N}}f(x) \vert \psi_{0} \vert ^{q}\,dx< 0. $$

So the proof of Step 1 is complete.

*Step* 2: the sequences $\{u_{b}^{i}\}$ ($i\in\{1,2\}$) contain strongly convergent subsequences.

By (4.1) and (4.2), we know that $\{ u_{b}^{i}\}$ ($i\in\{1,2\}$) are $(PS)$ sequences of the functionals $J_{b}(u)$. We claim that $\{u_{b}^{i}\}$ ($i\in\{1,2\}$) are bounded. In fact,

$$\begin{aligned} M&> J_{b} \bigl(u_{b}^{i} \bigr)=J_{b} \bigl(u_{b}^{i} \bigr)- \frac{1}{\theta p} \bigl\langle J_{b}^{\prime} \bigl(u_{b}^{i} \bigr),u_{b}^{i} \bigr\rangle \\ &\geq a \biggl(\frac{1}{p}-\frac{1}{\theta p} \biggr) \bigl\Vert u_{b}^{i} \bigr\Vert ^{p}- \biggl( \frac{1}{q}-\frac{1}{\theta p} \biggr)\lambda \vert f \vert _{\frac {p_{s}^{*}}{p_{s}^{*}-q}}S^{-\frac{q}{p}} \bigl\Vert u_{b}^{i} \bigr\Vert ^{q}, \end{aligned}$$

which shows that $\{u_{b}^{i}\}$ are bounded in $D^{s,p}(\mathbb {R}^{N})$ since $p>q>1$. With (4.1) and (4.2) at hand, we can see Lemma 2.7 as a special case to show that the sequences $\{u_{b}^{i}\}$ ($i\in\{1,2\}$) contain strongly convergent subsequences with $\{\widetilde{c_{b}},c_{b}\} < a(p_{s}^{*}-p)/(pp_{s}^{*})S^{{N}/{(ps)}}$. Hence there exist subsequences still denoted by themselves and $u^{i}\in D^{s,p}(\mathbb {R}^{N})$ such that $u_{b}^{i}\to u^{i}$ in $D^{s,p}(\mathbb {R}^{N})$ as $b\to0^{+}$ for $i\in\{1,2\}$. Therefore, $\forall\varphi\in C_{0}^{\infty}(\mathbb {R}^{N})$ we have

$$\begin{aligned} 0={}& \bigl(a+b \bigl\Vert u_{b}^{i} \bigr\Vert ^{(\theta-1)p} \bigr) \int_{\mathbb {R}^{2N}}\frac { \vert u_{b}^{i}(x)-u_{b}^{i}(y) \vert ^{p-2} (u_{b}^{i}(x)-u_{b}^{i}(y) ) (\varphi(x)-\varphi(y) )}{ \vert x-y \vert ^{N+ps}}\,dx\,dy \\ &{}- \int_{\mathbb {R}^{N}} \bigl\vert u_{b}^{i} \bigr\vert ^{p_{s}^{*}-2}u_{b}^{i}\varphi \,dx-\lambda \int_{\mathbb {R}^{N}}f(x) \bigl\vert u_{b}^{i} \bigr\vert ^{q-2}u_{b}^{i}\varphi \,dx \\ \to{}& a \int_{\mathbb {R}^{2N}}\frac{ \vert u^{i}(x)-u^{i}(y) \vert ^{p-2} (u^{i}(x)-u^{i}(y) ) (\varphi(x)-\varphi(y) )}{ \vert x-y \vert ^{N+ps}}\,dx\,dy \\ &{}- \int_{\mathbb {R}^{N}} \bigl\vert u^{i} \bigr\vert ^{p_{s}^{*}-2}u^{i}\varphi \,dx-\lambda \int_{\mathbb {R}^{N}}f(x) \bigl\vert u^{i} \bigr\vert ^{q-2}u^{i}\varphi \,dx \quad\text{as } b\to0^{+}, \end{aligned}$$

which shows that $u^{i}\in D^{s,p}(\mathbb {R}^{N})$ are solutions of (1.5) for $i\in\{1,2\}$.

*Step 3:* $J_{0}(u^{2})<0<J_{0}(u^{1})$.

Indeed,

$$J_{0} \bigl(u^{1} \bigr)=\lim_{b\to0^{+}}J_{b} \bigl(u_{b}^{1} \bigr)\geq\alpha>0 $$

and

$$J_{0} \bigl(u^{2} \bigr)=\lim_{b\to0^{+}}J_{b} \bigl(u_{b}^{2} \bigr)\leq -\frac{\lambda}{2q} \int _{\mathbb {R}^{N}}f(x) \vert \psi_{0} \vert ^{q}\,dx< 0. $$

Summing the above three steps, we see that $u^{1}$ and $u^{2}$ are two nontrivial solutions of (1.5). The proof is complete. □

## Asymptotic behavior as $a\searrow0^{+}$

In this section, we regard $a\in(0,1]$ as a parameter in problem (1.1) and analyze the convergence property. To do it, we have to prove that problem (1.1) admits at least two nontrivial solutions again. We introduce the following variational functional:

$$J_{a}(u)=\frac{a}{p} \Vert u \Vert ^{p}+ \frac{b}{\theta p} \Vert u \Vert ^{\theta p} -\frac{1}{p_{s}^{*}} \int_{\mathbb {R}^{N}} \vert u \vert ^{p_{s}^{*}}\,dx- \frac{\lambda}{q} \int _{\mathbb {R}^{N}}f(x) \vert u \vert ^{q}\,dx $$

to emphasize the independence of $a\in(0,1]$. In order to eliminate the influence of parameter $a>0$, we have the following lemma which is different from Lemma 2.5.

### Lemma 5.1

*There exists* $\lambda_{00}>0$ *such that the functional* $J_{a}(u)$ *satisfies the mountain*-*pass geometry around* $0\in D^{s,p}(\mathbb {R}^{N})$ *for any* $\lambda\in(0, \lambda_{00})$, *that is*:

(i) *there exist* $\alpha_{0},\rho_{0}>0$ *such that* $J_{a}(u)\geq\alpha >0$ *when* $\Vert u \Vert =\rho_{0}$ *and* $\lambda\in(0, \lambda_{00})$;
(ii) *there exists* $e_{0}\in D^{s,p}(\mathbb {R}^{N})$ *with* $\Vert e_{0} \Vert >\rho$ *such that* $J_{a}(e_{0})<0$.

### Proof

(i) It follows from (1.8) and Hölder’s inequality that

$$\begin{aligned} J_{a}(u)&\geq \Vert u \Vert ^{q} \biggl( \frac{b}{\theta p} \Vert u \Vert ^{\theta p-q}-\frac {1}{p_{s}^{*}}S^{-\frac{p_{s}^{*}}{p}} \Vert u \Vert ^{p_{s}^{*}-q} -\frac{\lambda }{q} \vert f \vert _{\frac{p_{s}^{*}}{p_{s}^{*}-q}}S^{-\frac{q}{p}} \biggr) \\ &\geq \biggl[\frac{bp_{s}^{*}S^{\frac{p_{s}^{*}}{\theta p}}(\theta p-q)}{\theta p(p_{s}^{*}-q)} \biggr]^{\frac{1}{p_{s}^{*}-\theta p}} \biggl\{ \frac{b(p_{s}^{*}-\theta p)}{\theta p(p_{s}^{*}-q)} \biggl[\frac {bp_{s}^{*}S^{\frac{p_{s}^{*}}{\theta p}}(\theta p-q)}{\theta p(p_{s}^{*}-q)} \biggr]^{\frac{\theta p-q}{p_{s}^{*}-\theta p}} - \frac{\lambda}{q} \vert f \vert _{\frac{p_{s}^{*}}{p_{s}^{*}-q}}S^{-\frac{q}{p}} \biggr\} . \end{aligned}$$

Therefore if we set

$$\begin{aligned} &\rho_{0}= \biggl[\frac{bp_{s}^{*}S^{\frac{p_{s}^{*}}{\theta p}}(\theta p-q)}{\theta p(p_{s}^{*}-q)} \biggr]^{\frac{1}{p_{s}^{*}-\theta p}}>0\quad \text{and}\\ & \lambda_{00}=\frac{bqS^{\frac{q}{p}}(p_{s}^{*}-\theta p)}{\theta p \vert f \vert _{\frac{p_{s}^{*}}{p_{s}^{*}-q}}(p_{s}^{*}-q)} \biggl[\frac{bp_{s}^{*}S^{\frac {p_{s}^{*}}{\theta p}}(\theta p-q)}{\theta p(p_{s}^{*}-q)} \biggr]^{\frac {\theta p-q}{p_{s}^{*}-\theta p}} >0, \end{aligned}$$

then there exists $\alpha_{0}>0$ such that $J_{a}(u)\geq\alpha_{0}>0$ when $\Vert u \Vert =\rho_{0}>0$ for any $\lambda\in(0,\lambda_{00})$.

(ii) Choosing $u_{0}\in D^{s,p}(\mathbb {R}^{N})\backslash\{0\}$, then since $\theta p< p_{s}^{*}$ and $f(x)$ is nonnegative one has

$$J(tu_{0})\leq\frac{a}{p}t^{p} \Vert u_{0} \Vert ^{p}+\frac{b}{\theta p}t^{\theta p} \Vert u_{0} \Vert ^{\theta p}-\frac{t^{p_{s}^{*}}}{p_{s}^{*}} \int_{\mathbb {R}^{N}} \vert u_{0} \vert ^{p_{s}^{*}}\,dx \to-\infty \quad\text{as } t\to+\infty. $$

Hence letting $e_{0}=t_{0}u_{0}\in D^{s,p}(\mathbb {R}^{N})\backslash\{0\}$ with $t_{0}$ sufficiently large, we have $\Vert e_{0} \Vert >\rho_{0}$ and $J_{a}(e_{0})<0$. The proof is complete. □

By Lemma 5.1, and the mountain-pass theorem in [31], a $(PS)$ sequence of the functional $J_{a}(u)$ at the level

5.1 $$ c_{a}:=\inf_{\gamma\in\Gamma}\max _{t\in[0,1]}J_{a} \bigl(\gamma(t) \bigr)\geq \alpha_{0}>0 $$

can be constructed, where the set of paths is defined as

$$\Gamma_{a}:= \bigl\{ \gamma\in C \bigl([0,1],D^{s,p} \bigl( \mathbb {R}^{N} \bigr) \bigr):\gamma(0)=0, J_{a} \bigl(\gamma(1) \bigr)< 0 \bigr\} . $$

In other words, there exists a sequence $\{u_{n}\}\subset D^{s,p}(\mathbb {R}^{N})$ such that

5.2 $$ J_{a}(u_{n})\to c_{a},\qquad J_{a}^{\prime}(u_{n})\to0\quad \text{as } n\to \infty. $$

The following two lemmas are very similar to Lemmas 2.6 and 2.7, respectively.

### Lemma 5.2

*There exists* $\lambda_{**}>0$ *such that the mountain*-*pass value satisfies*

$$c_{a}< c^{*}-C_{00}\lambda ^{\frac{p}{p-q}} \quad\textit{with } C_{00}=\frac{(\theta-1)(p-q)}{q\theta p} \biggl[\frac{(\theta p-q) \vert f \vert _{{p_{s}^{*}}/{(p_{s}^{*}-q)}}}{a(\theta-1)pS^{\frac{q}{p}}} \biggr]^{\frac{p}{p-q}} >0 $$

*for any* $\lambda\in(0,\lambda_{**})$, $c^{*}$ *and* *S* *are given by* (1.7) *and* (1.8), *respectively*.

### Lemma 5.3

*If* $\lambda\in(0,\lambda_{**})$, *any sequence satisfying* (2.2) *contains a strongly convergent subsequence whenever* $c_{a}< c^{*}-C_{00}\lambda^{\frac{p}{p-q}}$, *where* $c^{*}$ *is given by* (1.7).

### Remark 5.4

Since $a\in(0,1]$, always

$$c^{*}-C_{0}\lambda^{\frac{p}{p-q}}\geq c^{*}-C_{00} \lambda^{\frac{p}{p-q}}, $$

where $C_{00}>0$ is independent of *a*. Consequently, in addition to the proper adjustment of $\lambda_{**}$, the proof of Lemma 5.2 is exactly the same as that of Lemma 2.6. The above formula is applied to Eq. (2.6) to get a contradiction, hence we can prove Lemma 5.3.

In view of Sect. 3.2 and using Lemmas 5.1–5.3, we have the following proposition.

### Proposition 5.5

*Assume* (F), *then for any* $a,b>0$ *there exists a constant* $\lambda_{**}> 0$ *such that Eq*. (1.1) *has at least two nontrivial solutions*, $u_{a}^{11}$ *and* $u_{a}^{22}$, *satisfying*

$$J_{a} \bigl(u_{a}^{22} \bigr)< 0< J \bigl(u_{a}^{11} \bigr), \quad\forall \lambda\in(0, \lambda_{**}). $$

Now let $\lambda\in(0,\lambda_{**})$ and $b>0$ be fixed; we have the following.

### Proposition 5.6

*Let* $\{u_{a}^{11}\}$ *and* $\{u_{a}^{22}\}$ *be nontrivial solutions of* (1.1) *obtained in Proposition *5.5, *then there exist subsequences still denoted by themselves* $\{ u_{a}^{11}\}$ *and* $\{u_{a}^{22}\}$ *such that* $u_{a}^{ii}\to u^{ii}$ *in* $D^{s,p}(\mathbb {R}^{N})$ *as* $a\searrow0^{+}$ *for* $i\in\{1,2\}$, *where* $u^{11}$ *and* $u^{22}$ *are two nontrivial solutions of* (1.6).

### Proof

For any $a\in(0,1]$, there exists $M_{00}>0$ independent of *a* such that

$$\begin{aligned} c^{*}\leq {}&\biggl(\frac{1}{p}-\frac{1}{p_{s}^{*}} \biggr)S^{\frac{N}{ps}} \biggl(\frac{bS^{\frac{2\theta-1}{2}}+\sqrt{b^{2}S^{2\theta-1}+4 }}{2} \biggr)^{\frac{1}{\theta-1}} \\ &{}+b \biggl(\frac{1}{\theta p}-\frac{1}{p_{s}^{*}} \biggr)S^{\frac{N\theta}{ps}} \biggl( \frac{bS^{\frac{2\theta-1}{2}}+\sqrt{b^{2}S^{2\theta-1}+4 }}{2} \biggr)^{\frac{\theta}{\theta-1}} :=M_{00}< +\infty. \end{aligned}$$

Recalling Steps 1–3 in the proof of Theorem 1.5, we have the following facts.

*Fact* 1: there exist four constants independent of $a\in (0,1]$ such that

$$0< \alpha_{0}\leq c_{a}< M_{00}-C_{00} \lambda^{\frac{p}{p-q}} \quad\text{and}\quad {-}C_{00}\lambda^{\frac{p}{p-q}}\leq \widetilde{c_{a}}\leq -\frac{\lambda}{2q} \int_{\mathbb {R}^{N}}f(x) \vert \psi_{00} \vert ^{q}\,dx< 0, $$

where $\widetilde{c_{a}}=\inf\{J_{a}(u):u\in\overline{B}_{\rho_{0}}\}$.

*Fact* 2: the sequences $\{u_{a}^{ii}\}$ ($i\in\{1,2\}$) contain strongly convergent subsequences with $\{\widetilde{c_{a}},c_{a}\} < b(p_{s}^{*}-p)/(\theta pp_{s}^{*})S^{{N\theta}/{(ps)}}b^{{\theta}/{(\theta-1)}} S^{{\theta(2\theta-1)}/{2(\theta-1)}}$. Hence there exist subsequences still denoted by themselves and $u^{ii}\in D^{s,p}(\mathbb {R}^{N})$ such that $u_{a}^{ii}\to u^{ii}$ in $D^{s,p}(\mathbb {R}^{N})$ as $a\to0^{+}$ for $i\in\{1,2\}$. Therefore, $\forall\varphi\in C_{0}^{\infty}(\mathbb {R}^{N})$ we have

$$\begin{aligned} 0= {}&\bigl(a+b \bigl\Vert u_{a}^{ii} \bigr\Vert ^{(\theta-1)p} \bigr) \int_{\mathbb {R}^{2N}}\frac { \vert u_{a}^{ii}(x)-u_{a}^{ii}(y) \vert ^{p-2} (u_{a}^{ii}(x)-u_{a}^{ii}(y) ) (\varphi(x)-\varphi(y) )}{ \vert x-y \vert ^{N+ps}}\,dx\,dy \\ &{}- \int_{\mathbb {R}^{N}} \bigl\vert u_{a}^{ii} \bigr\vert ^{p_{s}^{*}-2}u_{a}^{ii}\varphi \,dx-\lambda \int_{\mathbb {R}^{N}}f(x) \bigl\vert u_{a}^{ii} \bigr\vert ^{q-2}u_{a}^{ii}\varphi \,dx \\ \to{}& b \bigl\Vert u^{ii} \bigr\Vert ^{(\theta-1)p} \int_{\mathbb {R}^{2N}}\frac { \vert u^{ii}(x)-u^{ii}(y) \vert ^{p-2} (u^{ii}(x)-u^{ii}(y) ) (\varphi(x)-\varphi(y) )}{ \vert x-y \vert ^{N+ps}}\,dx\,dy \\ &{}- \int_{\mathbb {R}^{N}} \bigl\vert u^{ii} \bigr\vert ^{p_{s}^{*}-2}u^{ii}\varphi \,dx-\lambda \int_{\mathbb {R}^{N}}f(x) \bigl\vert u^{ii} \bigr\vert ^{q-2}u^{ii}\varphi \,dx\quad \text{as } a\to0^{+}, \end{aligned}$$

which shows that $u^{ii}\in D^{s,p}(\mathbb {R}^{N})$ are solutions of (1.6) for $i\in\{1,2\}$.

*Fact* 3: $J_{00}(u^{22})<0<J_{00}(u^{11})$, where

$$J_{00}(u)=\frac{b}{\theta p} \Vert u \Vert ^{\theta p} - \frac{1}{p_{s}^{*}} \int_{\mathbb {R}^{N}} \vert u \vert ^{p_{s}^{*}}\,dx- \frac{\lambda}{q} \int _{\mathbb {R}^{N}}f(x) \vert u \vert ^{q}\,dx. $$

Therefore we know that $u^{11}$ and $u^{22}$ are two nontrivial solutions of (1.6). The proof is complete. □

### Proof of Theorem 1.7

In view of Sect. 3.2 and using Lemmas 5.1–5.3, we know that for any $a,b>0$ there exists a constant $\lambda_{**}> 0$ such that (1.1) has at least two nontrivial solutions, $u_{a}^{11}$ and $u_{a}^{22}$, satisfying

$$J_{a} \bigl(u_{a}^{22} \bigr)< 0< J \bigl(u_{a}^{11} \bigr),\quad \forall \lambda\in(0, \lambda_{**}). $$

Now we use Proposition 5.6 to obtain the desired result directly. The proof is complete. □

## Conclusion

This paper is concerned with the qualitative analysis of solutions of a nonlocal problem driven by the fractional *p*-Laplace operator. A key feature of this paper is the presence of the critical Sobolev exponent of Kirchhoff-type. We are interested both in the existence of solutions and in the multiplicity properties of the solutions. We also establish the convergence of solutions as the positive parameters converge to zero. There are obtained several very nice results and the variational arguments play a central role in the arguments developed in this paper. Finally, we obtain the threshold value by solving a quadratic algebra equation with one unknown which does not seem to have appeared in previous literature.
